# Enhancing holistic nursing care for sexual and gender minorities: A scoping review of global training initiatives

**DOI:** 10.4102/hsag.v31i0.3295

**Published:** 2026-04-23

**Authors:** Sibonelo Ndlovu, Neltjie C. van Wyk

**Affiliations:** 1 Department of Nursing Science, Faculty of Health Sciences, University of Pretoria, Pretoria, South Africa

**Keywords:** holistic nursing care, nurses, scoping review, sexual and gender minorities, training initiatives

## Abstract

**Background:**

Holistic nursing care integrates the physical, social, spiritual and psychological dimensions of well-being. Sexual and gender minority (SGM) groups experience healthcare disparities because of non-inclusive policies. To provide culturally competent care, nurses must receive training in addressing the needs of SGM individuals.

**Aim:**

This review synthesises evidence on the training nurses receive to deliver holistic care to SGM groups.

**Methods:**

Four electronic health databases – MEDLINE, Web of Science, ProQuest (Nursing & Allied) and EBSCOhost (CINAHL) – were searched for articles on nurses’ training for holistic care of SGM people, published between 2021 and 2025. Screening and reporting adhered to PRISMA flowchart guidelines, and Population, Concept and Context framework was utilised.

**Results:**

Three key themes emerged: the beneficial outcomes of healthcare training for nurses on SGM issues, factors hindering nurses from providing holistic care to SGM groups and characteristics of content in nursing education.

**Conclusion:**

Programmes aimed at equipping nurses to deliver holistic nursing care to SGM individuals require improvements in both their duration and delivery methods. It is imperative for higher education institutions to prioritise the provision of training on SGM issues for nurse educators, as this initiative is essential for substantially improving curriculum development. Collaboration among stakeholders can enhance healthcare outcomes for SGM groups.

**Contribution:**

This review can inform policies affecting SGM individuals, while helping develop a culturally competent workforce.

## Introduction

Nursing care that adopts a holistic perspective addresses the healthcare needs shaped by an individual’s physical, social, spiritual and psychological dimensions of well-being (Ambushe et al. 2023:1). This comprehensive approach to care is widely acknowledged as the standard for ensuring that patients’ healthcare needs are adequately met. However, individuals from sexual and gender minority (SGM) communities continue to encounter healthcare disparities because of the absence of inclusive policies in medical facilities that address their holistic healthcare needs (Menzer 2026:1). This highlights the necessity for healthcare facilities to revise policies and documentation to ensure that all gender identities and sexual orientations are recognised and protected (Fasullo et al. 2022:1087).

In evaluating the physical healthcare needs of SGM individuals, it is imperative for nurses to engage in sensitive listening and to eschew discriminatory assumptions (Medina-Martínez et al. 2021:8). Given that healthcare providers frequently lack experience with SGM populations, it is essential for patients to inform them about their specific healthcare requirements (Mitchell, Jacobs & McEwen 2023:7). Nurses should demonstrate a willingness to understand the healthcare needs of their SGM patients (Roach 2024:36). Maintaining confidentiality is crucial in providing holistic care to all patients, including those from SGM groups (Eliason, Chinn & ProQuest 2018:224). Nursing assessments should facilitate holistic care rather than be driven by mere curiosity (Cicero et al. 2024:87). Care that is imbued with bias continues to contribute to the discrimination of SGM individuals, particularly in the context of sexual healthcare (Casanova-Perez et al. 2022:278; Guzman et al. 2024:9).

Affirmative care practices that acknowledge and respect gender markers, such as the use of appropriate pronouns, may support individuals’ gender identity (Lippe et al. 2023:48). Such practices can foster inclusive environments for SGM individuals, who are often marginalised by the healthcare system (Cicero et al. 2024:91). The use of inclusive language and terminology may significantly enhance relationships between SGM individuals and healthcare professionals (Reeves et al. 2024:22; Sileo et al. 2022:8). Unfortunately, it appears that the use of preferred pronouns for patients from SGM groups is frequently neglected (Carroll et al. 2024:852). Such neglect may signal to the SGM community that they are not welcome in the hospital (Casanova-Perez et al. 2022:278).

Nurses should refrain from incorporating their personal spiritual beliefs into the care of SGM individuals (De Souza Maria De Aragão et al. 2022:4). It is frequently observed that nurses’ religious beliefs can adversely affect their attitudes towards SGM patients (Westwood, James & Hafford-Letchfield 2023:387). Conversely, patients often derive comfort from their own religious beliefs, which should be respected by healthcare providers Javier (2021:5). Patients from SGM groups require spiritual support, and healthcare providers have an obligation to ensure that their healthcare needs are adequately addressed (Sonneville 2023:204).

Psychological counselling and therapeutic support within sexual and reproductive healthcare may significantly enhance the well-being of SGM individuals (Seretlo, Smuts & Mokgatle 2024:8). A study conducted in Zambia by Mulavu et al. (2023:8) identified that SGM patients often experience psychological issues, including victimisation through physical threats and prejudice within healthcare environments. Jenkins et al. (2024:1) noted that SGM individuals residing in rural areas are more susceptible to depression and report receiving suboptimal healthcare services. Furthermore, Sileo et al. (2022:10) established a correlation between misconceptions about the SGM community and the prevalence of chronic illnesses, as exemplified by the erroneous belief that ‘HIV is gay’. According to Muwanguzi et al. (2023:7), nurses, through their own biases, may exacerbate the mental health challenges faced by SGM patients.

Providing culturally sensitive care to individuals from SGM communities is a fundamental obligation for nursing professionals (Stueben et al. 2024:1). Despite the expectation that nurses address the needs of a diverse patient population, some practitioners have reported feeling inadequately prepared to support individuals from SGM groups (Seretlo & Mokgatle 2023:4). This inadequacy may be attributed to a deficiency in educational programmes designed to enhance awareness of the healthcare needs of these marginalised communities (Argyriadis et al. 2023:2). Wang et al. (2025:6810) underscore the urgent necessity for initiatives that specifically address the healthcare requirements of these individuals.

Interest in the preparedness of nurses to provide care for SGM communities has been increasing across various regions, with readiness varying by location and institution. A study conducted by Muwanguzi et al. (2023:9) in Zambia indicated that nurses’ misconceptions regarding SGM lifestyles were altered following training. In the Philippines, research by Alibudbud (2024:1) advocated for the integration of SGM healthcare into nursing education. Conversely, in Switzerland, Nikitara et al. (2024:1) found that nurses felt inadequately prepared to care for SGM individuals despite having access to resources. Ancheta et al. (2025:4) observed that nurses demonstrated positive attitudes towards caring for SGM youth and expressed a desire for increased Lesbian, Gay, Bisexual, Transgender and Queer (LGBTQ+) education, although opinions differed regarding the collection of sexual orientation and gender identity data.

The researchers undertook a scoping review to delineate and map the existing body of knowledge, thereby illustrating how this study will contribute to its expansion. The motivation for this review arose from the documented healthcare disparities experienced by SGM individuals in the literature. Understanding the extent of nurses’ preparedness to care for SGM individuals on a global scale was essential. This understanding could facilitate the development of training initiatives to address the gap in care provided by nurses to SGM individuals.

## Methods

This scoping review aimed to delineate the current understanding of how nurses are trained to provide holistic care to SGM individuals. Conducting such a review helps to identify research gaps in this field (Banda, Mokgatle & Oladimeji 2025:5). The framework for scoping reviews by Arksey and O’Malley (2005) was utilised, encompassing (1) formulating the research question, (2) identifying relevant studies, (3) selecting studies, (4) organising the data and (5) compiling, summarising and presenting the findings. The protocol for this scoping review has not yet been registered.

In scoping reviews, there is neither an expectation nor a formal requirement to assess the risk of bias or the quality of evidence (Peters et al. 2021:3). This review adhered to the guidelines of the Preferred Reporting Items for Systematic Reviews and Meta-Analyses extension for scoping reviews (PRISMA-ScR), which employs a rigorous methodological approach to systematically identify, evaluate and summarise scientific literature in a transparent and reproducible manner. Utilising the PRISMA-ScR framework ensures comprehensive literature coverage, reduces bias and facilitates replication of the review process, thereby providing a robust foundation for drawing conclusions and making recommendations based on the collected data (Gottlieb et al. 2021:2).

### Stage 1: Identifying the research question

The research question posed was ‘What is known’ from the current literature regarding nurse training for holistic care of individuals from SGM groups? This review aimed to outline the existing knowledge on how nurses are educated to provide holistic care to SGM groups; assess the effects of training programmes on nurses; and examine the duration, delivery methods and content of the training. A broad approach should be maintained to ensure comprehensive coverage, allowing decisions to be made on setting parameters for large volumes of references once the scope of the field is better understood (Arksey & O’Malley 2005:23; Gottlieb et al. 2021:2). Therefore, scoping reviews do not employ structured questions as they aim to explore the breadth of research available on a topic, guided by a research question (Davies et al. 2024:2). Additionally, Gottlieb et al. (2021:2) suggested that researchers should consider the purpose and objectives of the review when formulating their research question, ensuring that the study has significant and relevant implications for educational policy, practice and/or further research. To assess whether the research question was suitable for this scoping review study, the researcher utilised the Population, Concept and Context framework for scoping reviews (see Table 1).

**TABLE 1:** Population, Concept and Context framework for determining the eligibility of the research question.

Criteria	Determinants
Population	Nurses from any part of the world, any group, all genders, regardless of their country and ethnic groups.
Concept	Training or induction of nurses to holistic care for people from sexual and gender minority groups. Literature focused on nurses training will be looked at in order to map the extent of existing research on the subject, and to identify the gaps in the knowledge base.
Context	Any sphere of healthcare where nurses interacted with people from SGM groups (hospitals and clinics offering primary care, tertiary care and rehabilitation).

SGM, sexual and gender minority.

### Stage 2: Identifying relevant studies

This scoping review included all studies published in peer-reviewed journals that concentrated on preparing or training nurses to deliver holistic care to individuals from SGM groups. As Arksey and O’Malley (2005:4) propose, the main goal of scoping the field is to comprehensively identify primary studies and reviews that can effectively address the research question. The studies were identified through a search of English-language literature published between 2021 and 2025, a timeframe selected based on the recent literature pertinent to the research question. A librarian was consulted to aid in developing the search strategy and identifying relevant databases (Carroll, Stokes & Darley 2021:3). MEDLINE Complete was the primary database used for this review because of its extensive coverage of healthcare publications. The search was confined to healthcare databases, including the Web of Science, ProQuest (Nursing and Allied) and EBSCOhost (CINAHL). Grey literature was excluded because it lacked sufficient details for the analysis process (Lee et al. 2024:2). The researcher employed key search terms such as ‘nurses’, ‘nursing staff’, ‘nursing practitioners’, ‘professional nurses’, ‘registered nurses’, ‘training’, ‘ongoing training’, ‘educational support’, ‘training programme’, ‘care’, ‘holistic care’, ‘inclusive care’, ‘comprehensive care’, ‘sexual and gender minority’, ‘LGBTIQI’, ‘lesbian’, ‘gay’, ‘transgender’ and ‘bisexual’. These terms were combined using ‘OR’ within each category and ‘AND’ across different categories.

#### Eligibility criteria

**Inclusion criteria:**

- Original research articles reporting information on the preparation of nurses in holistic care for people from SGM groups.
- The articles focused on nurses or similar roles in healthcare contexts.
- Articles published in peer-reviewed journals.
- Articles published in English.
- Articles published between 2021 and 2025.
- Articles involving participants recruited from all parts of the world.

**Exclusion criteria:**

- Non-English publications.
- Grey literature, including conference papers, theses and dissertations.
- Studies focused exclusively on all healthcare professionals other than nurses unless they provided data relevant to this scoping review.

### Stage 3: Study selection

The online software ‘Covidence’ was used for systematic screening to enhance efficiency and transparency.

**FIGURE 1:**
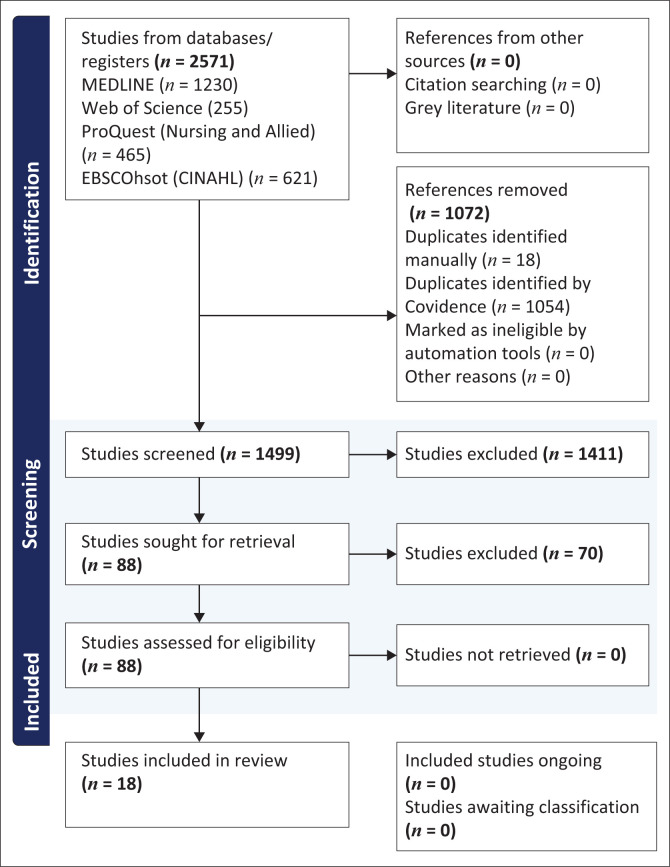
Outcome of the search strategy.

This tool streamlines the screening process, aiding the review of titles and abstracts for the final review stage (https://www.covidence.org/). As Arksey and O’Malley (2005:25) note, establishing a method to exclude irrelevant studies is essential. The inclusion criteria were holistic care provision, SGMs as care recipients, and nurses as care providers (Arksey & O’Malley 2005:26). The process of determining the inclusion and exclusion criteria was guided by identifying pertinent studies and collecting relevant data (Maluleke 2024:69). The selected publications were incorporated into the review, as depicted in the PRISMA-ScR flow diagram (Figure 1). After duplicates were removed, the researcher and supervisor assessed the titles and abstracts against the established criteria to identify eligible studies (Banda et al. 2025:4). Discrepancies were resolved through consensus-based dialogue. Sharma and Goyal (2023:55) recommended involving at least two reviewers, with conflicts resolved by consensus or a third reviewer. Studies meeting the criteria were uploaded to the Endnote software developed by Clarivate headquarters in Baar, Switzerland founded in 1996. Criteria were developed based on these research questions.

### Stage 4: Charting of data

The subsequent step involves ‘charting’ essential information from the primary research reports under review (Arksey & O’Malley 2005:26). As noted by Sharma and Goyal (2023:55), detailed data from the selected literature within the protocol ensures transparency. Data were collected in the following categories: author, title, journal, year and country, study aim, population, sampling method, sample size, research design and findings. This process ensured consistent data collection across the studies. The data extraction template was pilot-tested and revised after discussions with the study supervisor (Wanko Keutchafo et al. 2021:4). The researcher organised the data into a Microsoft Word spreadsheet with relevant headings (Godhwani et al. 2025:5). Table entries were checked for completeness (Andrew-Bassey et al. 2024:3). The supervisor reviewed the captured data, and the extraction was updated to enable the comprehensive abstraction of relevant data (Mapulanga & Dlungwane 2022). This facilitated an effective synthesis of findings regarding nurses’ training in holistic care for SGM individuals globally (see Table 2) (Lee et al. 2024:3).

**TABLE 2:** Extracted studies.

Author(s), title of the article, journal, year of publication & country	Aim of the study	Study population, sampling method and sample	Study design	Study findings
Brown et al. (2023), Ireland and US	The study aimed to identify LGBTQI health content within midwifery pre-registration programme and education and education best practices.	The sample included 29 academics (survey stage) and seven formed part of the final study.	1. Mixed methods design: (quantitative and qualitative methods). 2. Two-stage process: (1) online survey (36-item questionnaire) (2) semi-structured interviews. 3. Data analysis: thematic analysis using NVivo 12.	Academics prepared students for LGBTQ+ patients’ needs, avoiding heteronormative assumptions. Sessions covered same-sex parents and surrogacy while ensuring confidentiality for transgender patients and family safety.
Carmichael et al. (2024), US	The study examined the effect of transgender health education on nursing students’ knowledge and attitudes about caring for transgender and non-binary people (TGNB).	Senior nursing students (*n* = 71) completed pre-test surveys, with 46 final participants after exclusions.	The study used a pre-test and post-test design. A 2-h online TGNB healthcare education session served as the intervention. Participants completed TKABS-HP surveys before and after.	Participants’ TGNB care knowledge improved (*t*45 = 2.40; *P* = 0.02), scores rising from 9.63 (s.d. = 1.49) to 10.15 (s.d. = 1.01). Interpersonal comfort and human value remained unchanged. Terminology, health inequities, standards and nursing improved.
Cole et al. (2024), US	The study aimed to assess an LGBTQIA+ inclusivity training module for nursing students.	The study involved third-semester nursing students in a US programme. Of 42 initial participants, 30 completed all study phases.	This quasi-experimental study assessed nursing students’ knowledge and attitudes pre and post LGBTQIA+ inclusivity training. The intervention used Adobe Captivate™ computer-based simulation with inpatient and outpatient scenarios from NLN (2022) ACE+. Data was collected via surveys with Gay Affirmative Practice Scale and LGBTQIA+ knowledge assessment.	A moderate correlation existed between post-intervention GAP and knowledge assessment scores. The LGBTQIA+ computer-based simulation effectively improved nursing students’ attitudes and knowledge.
Fitze and Goodroad (2022), US	The aim was to develop a model for postsurgical genital affirming care using low-fidelity simulation and e-learning.	The study population included 50 surgical oncology unit nurses, with 30 nurses completing both pre-education survey, education programme, and post-education survey.	1. Staff nurses completed the education module during shift downtime over 8 weeks in 2021. The study was a quality improvement project: Educational intervention combined low-fidelity simulation, anatomic models and an electronic module. 2. Pre- and post-intervention surveys collected data on nurses’ knowledge and self-efficacy.	Knowledge scores rose from 3 to 5 (*p* < 0.001). Self-efficacy scores increased from 37.03 to 41.87 (*p* < 0.001). Low-fidelity models and a learning module improved nurse knowledge and self-efficacy for postsurgical genital affirmation care, effective for both experienced and novice nurses.
Jordan (2024), US	The aim was to increase nursing students’ knowledge of sexual orientation and gender identity (SOGI) in caring for LGBTQIA+ patients.	The study included third-semester prelicensure baccalaureate nursing students at a US university; 94 completed pre-intervention and 52 post-intervention surveys. The article lacks sampling technique details, although participants were students in a clinical practice course, suggesting convenience sampling.	The study used a flipped classroom approach with pre-recorded videos for self-instruction and classroom activities including group discussions and role-play. Learning outcomes were evaluated using pre- and post-test design.	Pre-intervention survey: 58% – 60% felt somewhat knowledgeable about SOGI, 18% – 22% very knowledgeable; 51% lacked gender identity courses; 35% felt equipped for LGBTQIA+ care. Post-intervention survey: 47% felt very knowledgeable about SOGI, 41% somewhat knowledgeable, 9.6% had little knowledge, 1.9% claimed expertise; 75% felt equipped to address LGBTQIA+ patient needs. Students felt uncomfortable asking SOGI questions. Patients found questions invasive. Role-play increased empathy for transgender patients.
Kaiafas and Kennedy (2021), US	This project aimed to improve care for LGBTQ patients in a military emergency department.	Through purposive convenience sampling, 42 nurses (63%) attended the first session and 39 (59%) the second session, with 72 total participants (36 pre/post-intervention unmatched).	The study evaluated nurses in a military emergency department through pre- and post-assessments. The intervention provided evidence-based education in two 30-min sessions. The AIM tool assessed participants’ knowledge, skills, openness, support and oppression awareness before and after.	1. Knowledge and Skills subscale scores increased post-intervention (*t* (70) = -3.33, *p* = 0.001), confirmed by Mann–Whitney *U* test (*U* = 368.5, *z* = -3.15, *p* = 0.002). The average score increased by 6.44%. 2. Oppression Awareness scores showed no difference pre- and post-intervention (*t* (70) = -0.93, *p* = 0.36; Mann–Whitney *U* = 554, *z* = -1.06).
Koch et al. (2021), US	The study aimed to improve nursing students’ ability to provide culturally sensitive care to transgender patients and address poor cultural intelligence among colleagues.	The study population comprised prelicensure nursing students in a second-degree Accelerated BSN programme’s foundations course in the Southeast US. Using convenience sampling, 29 of 72 participating students completed the post-test evaluation survey for final analysis.	The study used a descriptive post-test; mixed methods design in a foundations course for first semester nursing students. The survey included quantitative and qualitative questions to assess students’ learning and perceptions.	92.31% (*N* = 24) of students found the simulation helpful, with 41.67% (*N* = 10) rating it extremely helpful. Students showed improved awareness of transgender patient care through better pronoun use. The simulation would enhance patient communication and hospital policy inclusivity. Students suggested longer debrief discussions and pronoun guidance.
Maihle, Anderson and Von Sadovszky (2024), US	The aim of the study was to improve nurses’ knowledge and attitudes regarding LGBTQ patients and their families at a large, Midwestern paediatric hospital.	The study examined nurse clinical leaders in paediatric hospital clinics. Sixteen nurses were invited; 14 completed the pre-test and intervention, and 13 finished all stages. The final analysis included these 13 participants.	Participants were recruited via email and meetings to complete a pre-test in Redcap, followed by an educational module and post-test. The intervention consisted of a 30-min self-paced PowerPoint presentation adapted from the National LGBTQIA+ Health Education Centre’s module.	Knowledge: Overall, there was a significant increase in correct knowledge scores from pre-test (*M* = 13.62; s.d. = 1.71) to post-test (*M* = 14.85; s.d. = 0.38). Knowledge generally stayed consistent or increased across items. Major shifts in knowledge were related to understanding the experience and phases of ‘coming out’ and what it means to be bisexual. Some attitudes affirmed LGBT beliefs, while others remained unchanged. Participants opposed negative LGBT care attitudes. Competence and communication improved.
Mitchell and Potetz (2023), US	This study evaluated the effectiveness of a brief educational intervention on NP students’ competency in LGBTQ patient care.	Participants were nurses in family/adult-gerontology nurse practitioner programmes at a Midwestern university. Most (70%) were aged 25–34 and 90% female. From 30 initial participants, 13 completed both pre- and post-intervention surveys.	Participants completed a baseline survey on LGBTQ care knowledge, attended a health session with role-playing and completed follow-up after 6 weeks. Researchers compared pre- and post-intervention scores using mixed effect modelling.	Total competency scores increased to 5.78 (*p* < 0.001) and skills to 5.24 (*p* < 0.001). Knowledge rose to 5.87 (*p* = 0.014), while attitudinal awareness remained high (6.13–6.32, *p* = 0.89). Most participants (79%, *n* = 11) intended to change NP practice, with 43% (*n* = 6) changing RN practice.
Muwanguzi et al. (2023), Uganda	This study explored nurses’ attitudes towards caring for sexual and gender minorities (SGMs), specifically MSM and transgender persons, before and after training.	All participants had heard about MSM, but few had experience providing healthcare to MSM or transgender individuals. Initially, 25 nurses were approached, six declined, resulting in a final sample of 19 nurses.	This qualitative study used pre-post interviews to assess nurses’ attitudes towards sexual and gender minority patients before and after stigma reduction training. Analysis followed the ‘data analysis spiral’ of organising data, coding themes, interpreting and presenting findings.	Post-training reflections showed nurses gained better understanding of sexual orientation versus gender identity. Some suggested periodic training. While cultural beliefs persisted, nurses recognised their duty to provide professional care.
Priddle et al. (2025), Australia	This study explored LGBTIQ+ health content in Australian undergraduate nurse education among students, graduates and educators.	The study included nursing students (final year or graduated within 5 years), early career nurses (graduated within 5 years) and nursing educators teaching at Australian universities. The sample included two students, three nurses and four educators from five universities across three Australian states.	Using Cultural Safety theory for LGBTIQ+ communities, semi-structured interviews were conducted with students/graduates and educators via Zoom for 25–60 min. Reflexive thematic analysis used six steps to analyse experiences.	Students noted content gaps, while educators saw potential. Education used binary sex concepts, ignoring gender continuums. Educators differed on LGBTIQ+ teaching. LGBTIQ+ students self-protected in learning. Educators reflected on LGBTIQ+ content integration. Graduates noted poor LGBTIQ+ care preparation.
Rohde and Goode (2024), US	The study examined if simulation teaches nursing students gender-affirming care to improve knowledge and attitudes toward LGBTQIA+ patients.	One hundred seventy-one prelicensure nursing students enrolled in Introduction to Health Assessment participated in the simulation experience in the fall of 2022. The article lacks sampling method details, although all students participated, suggesting convenience sampling. Pre-activity survey: 98 participants completed it, 96 analysed. Post-activity had 20 (11.7%).	This study used simulation in nursing to teach care of transgender and gender non-conforming patients through pre-briefing, video viewing and debriefing. Surveys assessed LGBTQIA+ care knowledge using Likert and free-text questions, with statistical analysis of themes.	After simulation, students reported understanding transgender healthcare (70% strong, 30% some). All respondents felt confident providing gender-affirming care. 70% indicated strong confidence in therapeutic communication with transgender patients. Simulation effectively improved students’ confidence in caring for transgender and GNC patients, given their lack of prior knowledge.
Sherman et al. (2021), US	The study evaluated the Transgender Curriculum Integration Project’s impact on nursing students’ transgender health knowledge.	The study included 160 Johns Hopkins nursing students (2014–2015), with varying sample sizes across surveys. At the start of the programme: *n* = 80; after the introductory webinar in the first semester: *n* = 43. After completing the pre-licensure programme: *n* = 312. Of 160 students: 84% were 21–30 years; 75% white; 8.8% Latinx; 90% female.	Mixed methods with surveys and qualitative responses assessed students’ knowledge before and after TGD curriculum integration. Analysis identified themes and statistical differences.	TGD-specific content improved students’ gender sensitivity and care skills, although sensitivity remained low. Knowledge of TGD resources increased significantly. Students emphasised the importance of patient gender identity and requested more TGD content.
Tartavoulle and Laundry (2021), US	This study evaluated a programme to help nursing students provide culturally competent care for LGBQI+ patients.	The study included nursing students from six prelicensure schools in southeastern US. The sample of 1398 students was split between rural (71%) and urban (29%) areas, with no prior LGBTQI+ interventions.	A pre-test-post-test quasi-experimental design measured student attitudes and knowledge of LGBTQI+ individuals before and after the Advocacy Programme. Researchers assessed effects of a 2-h programme on nursing students without random assignment.	Attitudes improved on Genderism and Transphobia Scale and Homonegativity Scale (*S* = -19 350, *p* < 0.0001). LGBTQI+ policy knowledge increased from 61% to 79.4%, with rural students improving. Terminology knowledge rose from 65% to 79%, while treatment knowledge for transgender patients increased from 61% to 71.3%.
Tsusaki et al. (2023),US	The study assessed e-Learning feasibility for SGM healthcare, student recruitment’s impact, and LGBT-DOCSS effectiveness in measuring clinical preparedness for SGM populations.	The study population included family nurse practitioner (FNP) students. From 122 FNP students, 28 completed pre- and post-scale measures through voluntary sampling.	A pre-post-test study implemented an educational intervention on sexual and gender minority healthcare for nursing students. The LGBT Development of Clinical Skills Scale assessed preparedness. Content was delivered via e-Learning in nurse practitioner courses during August–December 2022.	The LGBT-DOCSS showed good internal consistency (alpha ≥ 0.80) pre- and post-intervention. Faculty found the content effective. Time constraints affected optional content completion. Post-test scores showed no change in bias awareness. The study proved feasible while identifying needed amendments.
Wang et al. (2023), Taiwan	This study aimed to develop and evaluate an online training programme for enhancing cultural competence in LGBT patient care among Taiwanese nurses and nursing students.	The study sampled nurses and student nurses aged 20+ from 42 nursing schools, 25 associations, and 97 long-term care facilities in Taiwan. Of 301 completed questionnaires, 201 were nurses (66.8%) and 100 student nurses (33.2%).	A pre-/post-test study was conducted, measuring outcomes before and after implementing an online training programme across nursing schools, associations and long-term care facilities.	Participants’ LGBT health knowledge increased post-test. LGB cultural competence improved and transgender competence increased. No differences in attitudes toward LGB or transgender post-programme. Limitations: discussion (14.7%), long videos (8.8%).
Webb and Zablocki (2023), US	This study explored nurse educators’ perceptions of teaching gender-affirming care to nursing students, focusing on LGBTQ+ patient care.	The study included nurse educators with 1 year of teaching experience in undergraduate nursing programmes. Purposive and snowball sampling was employed.	The study used a qualitative design to explore gender-affirming care teaching in nursing education and educators’ attitudes through open-ended questions.	Participants noted LGBTQ+ patient-centred care’s importance but had limited teaching opportunities. Most lacked LGBTQ+ terminology and transgender health knowledge. Barriers included time constraints, content overload, poor textbook coverage and discomfort from knowledge gaps or religious beliefs.
Ziegler et al. (2021), Canada	This study created an online toolkit focused on cultural humility for healthcare interactions with LGBTQI and Two-Spirit individuals.	Simulation experts and LGBTQI2S content experts designed games, with community members acting in virtual simulations.	The project used design thinking with LGBTQI2S community members and providers to develop a toolkit through team building and website creation. Testing incorporated user feedback from nursing students and faculty.	The team created a bilingual LGBTQI2S Cultural Humility Toolkit with simulations and resources for healthcare providers. LGBTQI2S community members acted in simulations, while testing with nursing students improved website diversity.

Note: Please see the full reference list of Ndlovu, S. & Van Wyk N.C., 2026, ‘Enhancing holistic nursing care for sexual and gender minorities: A scoping review of global training initiatives’, *Health SA Gesondheid* 31(0), a3295. https://doi.org/10.4102/hsag.v31i0.3295

ACE+, Advancing Care Excellence; AIM, Ally Identity measuring tool; BSN, Bachelor of Science in Nursing; GAP, Gay Affirmative Practice scale; GNC, Gender non-conforming; LGBT, Lesbian, Gay, Bisexual and Transgender; LGBT-DOCSS, Lesbian, gay, bisexual, transgender Development of Clinical skills scale; LGBTQI, Lesbian, Gay, Bisexual, Transgender and Queer; LGBTQI, Lesbian, Gay, Bisexual, Transgender, Queer/questioning and Intersex; LGBTQ, Lesbian, Gay, Bisexual, Transgender and Queer; LGBTQIA, Lesbian, Gay, Bisexual, Transgender, Queer/questioning, Intersex and Asexual; LGBTQIA+, Lesbian, Gay, Bisexual, Transgender, Queer/questioning, Intersex and Asexual (+) representing other identities; MSM, Men having sex with men; NP, Nurse Practitioner; RN, Registered Nurse; TGD, Transgender and gender diverse; TKABS-HP, transgender knowledge, attitudes and beliefs scale for health care professionals; US, United States.

### Ethical considerations

Ethical clearance to conduct this study was obtained from the Faculty of Health Sciences Research Ethics Committee of the University of Pretoria (No. 338/2025).

## Results

### Stage 5: Collating, summarising and reporting the results

The evaluation of data gathered through the data extraction framework sheds light on existing research on the training of nurses in providing holistic care to SGM individuals. It also highlights the knowledge gaps in this area of study (Carroll et al. 2021:4). The outcomes of the search strategy and selection processes are presented in the PRISMA-ScR diagram (Sharma & Goyal 2023:55). Refer to Figure 1.

#### Characteristics of the selected publications

The scoping review included 18 publications, with the publication years ranging from 2021 to 2025.

Significantly 2023 saw the highest number of publications, accounting for 33.3% of all publications. The majority of the studies were conducted in the United States (US), which contributed the most publications (*n* = 13). Other countries, including Australia, Canada, Ireland, Uganda and Taiwan, each provided one study (5.5%). The most common study designs were pre- and post-intervention (*n* = 8), followed by qualitative (*n* = 3), quasi-experimental (*n* = 2), mixed method (*n* = 2), survey (*n* = 1), non-randomised (*n* = 1) and project-utilised (*n* = 1). The sample sizes were mentioned in 17 publications. Regarding the study population, student-focused studies had the largest number of participants, with 10 (55.5%), compared to nurses with 3 (16.6%), nursing academics with 2 (11.1%), nursing clinical leaders with 1 (5.5%), a combined group of nursing students, nursing academics and nurses with 1 (5.5%), and an unspecified group with 1 (5.5%).

#### Content analysis

Thematic content analysis was employed to interpret narrative accounts from selected studies, adhering to Braun and Clarke’s (2006) six-step framework: (1) familiarisation, (2) coding, (3) generating themes, (4) reviewing themes, (5) defining and naming themes and (6) report writing. The researchers engaged deeply with the data by repeatedly reading the extracted articles. Relevant sections from these articles were transcribed and organised into a spreadsheet, with each segment being individually analysed and inductively coded (Braun & Clarke 2006:87). According to Oke and Sibomana (2025:3), the literature should be independently coded and categorised into main themes by two individuals, with disagreements resolved through discussions and unresolved issues referred to a third reviewer for adjudication. To mitigate personal biases that might influence data interpretation, the researcher maintained a reflective journal during the analysis phase (Corrigan et al. 2025:5). Rather than focusing on the quality of each study individually, the review provides a comprehensive summary of the evidence by compiling findings and presenting an organised overview (Banda et al. 2025:5). Arksey and O’Malley (2005:27) note that unlike a systematic review, a scoping study does not aim to ‘synthesise’ evidence or combine findings. While a scoping study requires an analytical framework or thematic structure for a narrative overview of the literature, it does not evaluate the ‘weight’ of the evidence on specific interventions or policies.

**Theme 1: Beneficial outcomes of healthcare training for nurses in sexual and gender minority issues:** Among the 18 publications reviewed, 12 emphasised knowledge retention as the primary outcome. Of these, seven employed pre- and post-test designs, three used quasi-experimental pre- and post-test designs, one adopted a mixed method approach, and one utilised a survey design (see Table 3). These studies, conducted across various geographical regions and settings, showed methodological diversity. All reported improved scores following the educational interventions. Three studies (Koch et al. 2021; Mitchell et al. 2023; Wang et al. 2023) highlighted enhancements in participants’ cultural competency post-training, including increased awareness and empathy towards transgender patient care, as well as greater mindfulness in language use and confidence in pronoun application. Seven studies observed a positive shift in nurses’ attitudes towards LGBTQ+ healthcare needs after training (Cole et al. 2024; Maihle, Anderson & Von Sadovszky 2024; Mitchell et al. 2023; Muwanguzi et al. 2023; Rohde & Goode 2024; Tartavoulle & Laundry 2021). Webb and Zablocki (2023) found that nurse educators recognise the importance of LGBTQ+ patient-centred care. Despite personal beliefs regarding SGM, a study by Muwanguzi et al. (2023) in Ghana revealed that nurses acknowledged their professional duty to provide care irrespective of differences after training. Two studies by Koch et al. (2021) and Rohde et al. (2024) indicated that student nurses developed an interest in patients’ gender identity and empathy towards marginalised groups following the training session.

**TABLE 3:** Characteristics of 18 publications in the scoping review.

Characteristics	Number	%
**Year of publication**		
2021	5	27.7
2022	1	5.5
2023	6	33.3
2024	5	27.7
2025	1	5.5
**Countries**		
Australia	1	5.5
Canada	1	5.5
Ireland and US	1	5.5
Taiwan	1	5.5
Uganda	1	5.5
US	13	72.2
**Study population**		
Nursing students	10	55.5
Nursing academics	2	11.1
Nursing clinical leaders	1	5.5
Nurses	3	16.6
Nursing students, nursing academics and nurses	1	5.5
Not specified	1	5.5
**Study design**		
Mixed method design	2	11.1
Quasi-experimental design	2	11.1
Pre- and post-intervention design	8	44.4
Qualitative research design	3	16.6
Non-randomised design	1	5.5
Survey study design	1	5.5
Project utilised design	1	5.5

US, United States.

**Theme 2: Factors impeding nurses in the provision of holistic care to sexual and gender minority groups:** Three distinct studies conducted by Muwanguzi et al. (2023); Priddle, Crawford and Power (2025) and Tsusaki, Mullassery and Padmavathy (2024) have underscored the challenges associated with delivering holistic care to individuals from SGM communities. Two of these investigations utilised qualitative research methodologies, while the third employed a non-randomised design (see Table 3). The findings revealed that time constraints hindered students from engaging with optional LGBTQ+ content. Ugandan nurses identified legal restrictions and religious beliefs as significant barriers to providing care to SGM patients. An Australian study highlighted negative attitudes, noting that nursing education is predominantly shaped by heteronormative norms and practices, with limited inclusion of LGBTQ+ content. Frequently, LGBTQ+ material is integrated into other subjects and often designated as ‘optional’. Educators reported uncertainty and a lack of consensus regarding the quantity, delivery and evaluation of LGBTQ+ content within the curriculum.

**Theme 3: Characteristics of sexual and gender minority content in nursing education:** Nurse educators have identified a deficiency in opportunities to instruct nursing students in patient-centred care specifically tailored to LGBTQ+ individuals (Table 3) (Webb et al. 2023). The majority of participants reported a lack of formal training in caring for members of the LGBTQ+ community (Priddle et al. 2025). In research conducted by Brown et al. (2023), educators expressed a willingness to incorporate LGBTQ+ curricula into nursing education and acknowledged the diversity and specific needs of LGBTQ+ individuals in midwifery education, such as surrogacy and same-sex couples. Some universities have appointed ‘LGBTQ+ champions’ to assist in the development of their training programmes (Brown et al. 2023). Participants proposed various strategies to enhance LGBTQ+ content in nursing curricula, including an understanding of their historical experiences (Priddle et al. 2025). To improve care, a toolkit was successfully developed to support nurses and other healthcare professionals in promoting cultural humility through self-reflection, knowledge acquisition, and practical application (Ziegler et al. 2021). A study by Koch et al. (2021) found that most students recommended extending debriefing sessions to enhance practice. Many students valued the opportunity to learn about LGBTQ+ healthcare needs through simulation, as they had previously lacked knowledge in this area. Additionally, students noted that simulations improved their communication with LGBTQ+ patients and strengthened their ability to advocate for more inclusive hospital policies. Research by Priddle et al. (2025) revealed differing opinions among participants regarding the LGBTQ+ content. While students and recent graduates viewed the content as reinforcing deficit narratives, educators noted some positive developments in curricula. Studies by Sherman et al. (2021) and Rohde and Goode (2024) found that students expressed a need for more LGBTQ+ content in the curriculum, highlighting their interest in expanding their knowledge in this field.

## Discussion

In response to a call for a deeper understanding of how well-prepared nurses are to provide holistic care to individuals from SGM communities, this scoping review aimed to map the global literature on training nurses to deliver such care to people in SGM groups. Screening and reporting adhered to PRISMA flowchart guidelines, and a narrative synthesis was conducted. Population, Concept and Context framework for scoping reviews was utilised. The picture emerging from the conducted analysis explains a dire need for nurse training initiatives in healthcare needs for SGM people.

The review process involved examining 2571 publications for potential inclusion and ultimately selecting 18 articles from various databases that met the inclusion criteria. The selected studies encapsulated the essence of this scoping review by illuminating the current understanding of nurses’ training in holistic care for SGM groups. The analysis revealed three themes that elucidate the current state of knowledge in this area of research. The researcher used these themes to identify gaps and areas that warrant further investigation.

The results of this review provide evidence that training nurses can positively impact the well-being of SGM individuals. Acquiring knowledge led to a change in nurses’ perspectives regarding the lifestyles of individuals in SGM communities. Cultural competency training for SGM individuals has resulted in improved knowledge and attitudes. This aligns with the findings of Elertson and McNiel (2021:2239), where most students reported heightened awareness of health disparities affecting LGBTQ individuals, a better understanding of supportive health services, and an enhanced ability to engage with LGBTQ individuals in health promotion. The participants demonstrated increased empathy and mindfulness in language use, particularly pronoun usage, when caring for transgender individuals. Soled et al. (2022:5); Schultz et al. (2025:957) emphasised the essential role of language in delivering culturally sensitive and person-centred care. Employing a suitable and inclusive language can foster trust, enhance patient experience, and lead to improved health outcomes. Nurse educators have acknowledged the urgent need for SGM content in nursing education to enhance healthcare experience. Similarly, Carney and Baser (2026:11) argued that using immersive, competency-based simulations can successfully incorporate SGM health topics into nursing education before licensure, promoting supportive care practices and filling significant gaps in conventional curricula. In a Ugandan study, nurses demonstrated tolerance after training by committing to caring for patients despite cultural beliefs and laws opposing SGM individuals. According to Wang et al. (2025:6803), the organisational climate, especially in traditional or religious hospitals, along with a lack of training programmes, is recognised as an obstacle to delivering culturally competent care to individuals from SGM groups.

Three studies underscored the difficulties in delivering holistic care to SGM groups, primarily because of systemic and legal challenges. One significant issue is the legal restrictions in Uganda, where SGMs are not recognised and same-sex relationships are banned. A study from Nigeria by Ogunbajo et al. (2021:1696) echoes this view, revealing that SGM individuals often encounter violence and mistreatment from both public and law enforcement because of their sexual orientation. Another limitation is the lack of time to address LGBTQ+ content as nurse educators are constrained by demanding schedules. Additionally, there is resistance to incorporating LGBTQ+ topics because of entrenched heteronormative practices. The module is optional and is not deemed essential. Nye et al. (2024:205) point out that faculty perceptions of LGBTQ+ health are often influenced by a ‘deficit narrative’, which highlights health risks and adverse healthcare outcomes. These beliefs are typically assessed using normative or heteronormative tools.

Nurse educators have observed a lack of opportunity to integrate LGBTQ+ topics into nursing education. Most respondents indicated that they had not received formal training in delivering education and care to LGBTQ+ individuals. As noted by Gedzyk-Nieman and Hand (2023:410), nursing faculty often bear the responsibility of teaching students about LGBTQ+ issues despite the lack of specialised expertise in this area. Another study pointed out that nurse educators are willing to provide nursing students with LGBTQ+ curricula, recognising the unique diversity and needs of LGBTQ+ individuals in midwifery education, including topics such as surrogacy and same-sex couples. Hodges, Seibenhener and Young (2021:116) support this view, calling for a fundamental shift in nursing education by revising nursing curricula to include concepts pertinent to the LGBTQ community.

Several universities have appointed ‘LGBTQ+ champions’ to assist in developing their training programmes.

Participants proposed various methods to enhance LGBTQ+ content in nursing curricula, such as gaining insight into the community’s historical experiences. Wang et al. (2025:11) advocated implementing relevant training programmes and creating an LGBT-friendly environment by equipping nurses with the necessary insights and knowledge. To improve care, a toolkit was developed to support nurses and other health care workers in promoting cultural humility through self-examination, learning and practical implementation. Goodall and Wofford’s (2022:1) integrative review underscores the importance of strategies suitable for LGBTQ+ curricula or programmes to foster cultural humility. Most students recommended extending the debriefing sessions to enhance their practice. Stueben et al. (2024:1) advocated future implementations to include a group of trained standardised participants and to allocate sufficient time for pre-briefing, scenario facilitation and debriefing.

Many students valued the opportunity to learn about LGBTQ+ healthcare needs through simulation, as they previously lacked knowledge in this area. Additionally, students noted that simulations improved their communication with LGBTQ+ patients and strengthened their ability to advocate more inclusive hospital policies. The findings of Cortez (2022:31) indicated that students experienced an enhancement in their knowledge and confidence when it came to providing care for LGBTQ+ patients after completing the modules and simulation.

Participants held varied opinions on the LGBTQ+ content. While students and recent graduates viewed the content as perpetuating deficit narratives, educators observed positive changes in the curricula. Nwankwo and Nana (2024:303) found in their research that SGM individuals were seen as opposing both Christianity and African cultural values. Many students expressed a desire for more LGBTQ+ content in their curriculum, highlighting their interest in expanding their knowledge. Lemus Celin et al. (2025:1) phenomenological study underscored the importance of embracing the diversity of SGM individuals and introducing educational activities tailored to their healthcare needs.

### Strengths of the study

This review demonstrates several strengths and weaknesses. A notable strength is the incorporation of a broad spectrum of cultural perspectives, enabled by the diverse countries and cultures represented in the research.

Furthermore, the review’s methodology is robust, characterised by clear inclusion criteria, the utilisation of four healthcare databases in the search strategy, and the involvement of two reviewers to ensure a comprehensive screening process for all eligible studies.

### Limitations of the study

In accordance with standard procedures for scoping reviews, the inclusion and exclusion criteria were intentionally broad to capture a wide range of data. All decisions were reached through consensus to ensure consistent application of the criteria by reviewers. The search strategy employed specific terms to locate articles on training nurses for SGM care. However, articles discussing healthcare professional training that included nurses might have been overlooked. Additionally, articles not in English and grey literature were excluded, which could limit representation from fields where journals are not the primary scholarly medium.

### Future research

Existing studies have concentrated on brief training programmes for nurses who care for SGM communities. Longitudinal research could monitor shifts in nurses’ attitudes over time. It is important for research to explore how organisations can assist nursing education in incorporating SGM curriculum as a mandatory module.

## Conclusion

This scoping review examined nurse training programmes and their outcomes in global literature. Programmes designed for nurses providing holistic care to SGM individuals require enhancements in both duration and delivery methods. Nursing education leaders and administrators must sustain these initiatives and assess their effectiveness. Supporting educators with training on SGM issues will improve curriculum development. Collaboration among stakeholders in implementing these programmes can lead to better healthcare outcomes for SGM communities.
